# Supplementary material for: Determinants of Depressive Symptoms in People Living with HIV: Findings from a Population-Based Study with a Gender Perspective
Source: Int J Environ Res Public Health. 2023 Feb 19;20(4):3687. doi: 10.3390/ijerph20043687 (PMC9964424; doi:10.3390/ijerph20043687)
Supplement: Supplementary file 1 [file ijerph-20-03687-s001.zip › ijerph-2161473-supplementary.pdf]

Supplementary material

- Multiple imputation processing

We used variables as predictors when they were correlated with the exposure levels and/or with the probability of the exposure being missing (absolute correlation value>0.1) (Supplementary Table S1). For each predictor, the proportion of observed values among the non-observed values was no greater than 25%. After imputation, we conducted the following diagnostics. We compared imputed and observed data using density and stripplots of van Buuren and Greenacre [61]. These types of comparison were only done when there were more than 5% of non-observed values. Numerically, we checked that variables had 1) an absolute difference between means of the observed and imputed values smaller than 2 standard deviations; and 2) a ratio of variances of the observed and imputed values between 0.5 and 2. For categorical variables, we ensured the p-value of the chi-squared test between imputed and non-imputed values was >0.05.

Table S1. Predictor's covariance matrix.

	Gender	Age category	Born abroad	Level of education	Occupation	Income	Sexual attraction	Recent sex work	Satisfaction with sex life	Alcohol	Diagnosed last 12	Mode of transmission	Substance use	Nicotine dependence	Sexual partners last 6	No. sexual partners	Sexualized drug use	Serologic disclosure	Been treated differently	Denied care	Stigma and	Social	Satisfaction social role	Hours dedicated to	Hours spent caring for	QOL - physical	QOL - mental	Cognitive function	No. predictors	No. missing
Gender	0	0	0	1	1	1	1	0	0	1	0	1	1	0	1	1	1	0	0	0	0	0	0	0	1	1	0	0	12	8
Age category	0	0	0	0	0	0	0	0	0	0	0	0	0	0	0	0	0	0	0	0	0	0	0	0	0	0	0	0	0	0
Born abroad	0	0	0	0	0	0	0	0	0	0	0	0	0	0	0	0	0	0	0	0	0	0	0	0	0	0	0	0	0	0
Level of education	1	1	1	0	1	1	1	0	0	1	0	0	1	1	0	1	1	0	0	0	0	0	1	0	1	1	0	0	14	17
Occupation	1	1	1	1	0	1	1	0	1	1	1	0	1	0	1	1	1	1	0	0	1	0	1	1	1	1	1	1	21	22
Income	1	0	0	1	1	0	1	0	0	1	0	0	0	0	0	1	1	0	0	0	1	1	1	0	1	1	1	1	14	85
Sexual attraction	1	1	1	1	1	1	0	1	0	1	0	1	1	0	1	1	1	0	0	0	0	0	0	0	1	1	0	0	15	18
Recent sex work	0	1	1	0	0	0	1	0	0	0	1	0	1	0	0	1	1	0	0	0	0	0	1	0	0	0	0	0	8	20
Satisfaction with sex life	1	1	0	1	1	1	1	0	0	1	0	1	1	0	1	1	0	0	1	1	1	1	1	0	0	1	1	1	19	98
Alcohol dependence	1	1	0	1	1	1	1	0	0	0	0	0	1	0	1	1	1	0	0	0	0	0	0	0	1	0	0	0	11	17
Diagnosed in the last 12 months	0	1	1	1	1	0	0	1	0	0	0	0	0	0	0	1	0	1	0	0	0	0	0	0	0	0	0	1	8	133
Mode of transmission	1	1	0	0	1	0	1	0	0	0	0	0	1	1	1	1	1	1	0	0	0	1	0	0	0	0	0	0	11	55
Substance use	1	1	1	1	1	0	1	1	0	1	0	1	0	1	1	1	1	0	0	0	1	1	0	0	1	1	0	0	17	52
Nicotine dependence	0	1	1	1	0	0	0	0	0	0	0	1	1	0	0	0	1	1	0	0	1	1	1	0	0	1	1	1	13	19
Sexual partners in the last 6 months	1	1	0	0	1	0	1	0	1	1	0	1	1	0	0	1	1	0	0	0	0	0	0	0	0	1	0	0	11	31
No. of sexual partners	1	1	1	1	1	1	1	1	0	1	1	1	1	0	1	0	1	0	0	0	0	0	0	0	1	1	0	0	16	31

Sexualized drug use	1	1	1	1	1	1	1	1	0	1	0	1	1	1	1	1	0	0	0	0	0	1	1	0	0	1	1	1	19	17
Serologic disclosure	0	0	1	0	1	0	0	0	0	0	1	1	0	1	0	0	0	0	1	0	1	0	0	0	0	0	0	0	7	24
At your health centre: had been treated differently	0	0	0	0	0	0	0	0	1	0	0	0	0	0	0	0	0	1	0	1	1	1	1	0	0	1	1	1	9	16
At your health centre: had denied care or delayed treatment	0	0	0	0	0	0	0	0	1	0	0	0	0	0	0	0	0	0	1	0	1	1	1	0	1	1	1	1	9	16
Stigma and discrimination	0	0	0	0	1	1	0	0	1	0	0	0	0	1	0	0	0	1	1	1	0	1	1	0	1	1	1	1	13	17
Social isolation	0	0	0	0	0	1	0	0	1	0	0	1	1	1	0	0	1	0	1	1	1	0	1	0	0	1	1	1	13	1
Satisfaction with social role	0	0	0	0	0	0	0	0	0	0	0	0	0	0	0	0	0	0	0	0	0	0	0	0	0	0	0	0	0	0
Hours dedicated to leisure	0	1	0	1	1	0	0	0	0	0	0	0	0	0	0	0	0	0	0	0	0	0	0	0	0	0	0	0	3	261
Hours spent caring for others	1	0	0	1	1	1	1	0	0	1	0	0	1	0	0	1	0	0	0	1	1	0	0	1	0	1	0	0	12	225
Quality of life - physical	0	0	0	0	0	0	0	0	0	0	0	0	0	0	0	0	0	0	0	0	0	0	0	0	0	0	0	0	0	0
Quality of life - mental	0	0	0	0	0	0	0	0	0	0	0	0	0	0	0	0	0	0	0	0	0	0	0	0	0	0	0	0	0	0
Cognitive function	0	0	0	0	0	0	0	0	0	0	0	0	0	0	0	0	0	0	0	0	0	0	0	0	0	0	0	0	0	0

Table S2. Differences between non-imputed and imputed data in the included predictors.

Variables	Imputed N=1060	Non-imputed N=1060	*p* value
Gender, n (%)			0.999
Men	833 (78.58)	827 (78.02)	
Women	192 (18.11)	190 (17.92)	
Transgender	35 (3.3)	35 (3.3)	
Missing	0 (0)	8 (0.75)	
Age category, n (%)			1
≤39	236 (22.26)	236 (22.26)	
40-59	615 (58.02)	615 (58.02)	
≥60	209 (19.72)	209 (19.72)	
Born abroad (yes), n (%)	354 (33.4)	354 (33.4)	1
Level of education, n (%)			0.974
Without or primary education	236 (22.26)	228 (21.51)	
Secondary school	280 (26.42)	278 (26.23)	
Higher education	544 (51.32)	537 (50.66)	
Missing	0 (0)	17 (1.6)	
Occupation, n (%)			0.999
Currently working	602 (56.79)	593 (55.94)	
Not currently working	168 (15.85)	164 (15.47)	
Retired	161 (15.19)	158 (14.91)	
Housework	25 (2.36)	23 (2.17)	
Be on leave	104 (9.81)	100 (9.43)	
Missing	0 (0)	22 (2.08)	
Income, n (%)			0.989
No income	97 (9.15)	90 (8.49)	
<1000 €	391 (36.89)	365 (34.43)	
1001-2000 €	425 (40.09)	389 (36.7)	
>2001 €	147 (13.87)	131 (12.36)	
Missing	0 (0)	85 (8.02)	
Sexual attraction, n (%)			0.973
Heterosexual	417 (39.34)	405 (38.21)	
Homosexual	530 (50)	526 (49.62)	
Bisexual	113 (10.66)	111 (10.47)	
Missing	0 (0)	18 (1.7)	
Recent sex work, n (%)			1
No	1022 (96.42)	1003 (94.62)	
Yes	38 (3.58)	37 (3.49)	
Missing	0 (0)	20 (1.89)	
Quality of life - physical, median [IQR]	47.47 [37.11-66.42]	47.47 [37.11-66.42]	1
Quality of life - mental, median [IQR]	56.76 [36.03-72.48]	56.73 [36.03-72.48]	1
Cognitive function, median [IQR]	50.50 [32.65-64.20]	50.50 [32.65-64.20]	1
Overall satisfaction with sex life, n (%)			0.627
Satisfied	874 (82.45)	802 (75.66)	
Unsatisfied	186 (17.55)	160 (15.09)	
Missing	0 (0)	98 (9.25)	
Alcohol dependence, n (%)			0.98

Non-drinker	282 (26.6)	274 (25.85)	
Low risk drinker	584 (55.09)	579 (54.62)	
High risk drinker	194 (18.3)	190 (17.92)	
Missing	0 (0)	17 (1.6)	
Diagnosed in the last 12 months (yes), n (%)			1
No	983 (92.74)	860 (81.13)	
Yes	77 (7.26)	67 (6.32)	
Missing	0 (0)	133 (12.55)	
Mode of transmission, n (%)			0.589
PWID	211 (19.91)	190 (17.92)	
MSM	615 (58.02)	605 (57.08)	
HHTX	87 (8.21)	69 (6.51)	
MHTX	147 (13.87)	141 (13.3)	
Missing	0 (0)	55 (5.19)	
Substance use, n (%)			0.992
Cluster 1	823 (77.64)	784 (73.96)	
Cluster 2	145 (13.68)	136 (12.83)	
Cluster 3	92 (8.68)	88 (8.3)	
Missing	0 (0)	52 (4.91)	
Nicotine dependence, n (%)			0.996
Non smoker	607 (57.26)	598 (56.42)	
Low nicotine dependence	210 (19.81)	206 (19.43)	
Medium-high nicotine dependence	243 (22.92)	237 (22.36)	
Missing	0 (0)	19 (1.79)	
Sexual partners in the last 6 months, n (%)			0.998
None	203 (19.15)	195 (18.4)	
Steady partner and occasional partner	165 (15.57)	163 (15.38)	
Only steady partner	418 (39.43)	405 (38.21)	
Only occasional partners	274 (25.85)	266 (25.09)	
Missing	0 (0)	31 (2.92)	
Number of sexual partners, n (%)			0.988
Tercil 1 [0-3]	621 (58.58)	600 (56.6)	
Tercil 2 [3-7]	183 (17.26)	174 (16.42)	
Tercil 3 [7-360]	115 (10.85)	114 (10.75)	
Not applicable	141 (13.3)	141 (13.3)	
Missing	0 (0)	31 (2.92)	
Sexualized drug use, n (%)			0.999
No	578 (54.53)	568 (53.58)	
Once in a lifetime	236 (22.26)	230 (21.7)	
Last year	106 (10)	105 (9.91)	
Last month	140 (13.21)	140 (13.21)	
Missing	0 (0)	17 (1.6)	
Serologic disclosure, n (%)			1
None	169 (15.94)	165 (15.57)	
Less than half	695 (65.57)	679 (64.06)	
All or almost all	196 (18.49)	192 (18.11)	
Missing	0 (0)	24 (2.26)	
At your health centre: had been treated differently (yes), n (%)			0.964

No	849 (80.09)	838 (79.06)	
Yes	211 (19.91)	206 (19.43)	
Missing	0 (0)	16 (1.51)	
At your health centre: had denied care or delayed treatment (yes), n (%)			1
No	922 (86.98)	909 (85.75)	
Yes	138 (13.02)	135 (12.74)	
Missing	0 (0)	16 (1.51)	
Stigma and discrimination, median [IQR]	10.00 [8.00-26.00]	10.00 [8.00-26.00]	0.908
Social isolation, median [IQR]	43.20 [34.00-64.65]	43.2 [34.00-64.66]	0.988
Satisfaction with social role, median [IQR]	47.20 [38.90-49.20]	47.20 [38.90-49.20]	1
Hours dedicated to leisure, mean (SD)	10.00 [0.00-58.10]	10.00 [0.00-50.00]	0.208
Hours spent caring for others, mean (SD)	0.00 [0.00-50.00]	0.00 [0.00-48.00]	0.634

Note. The presented results are from a random of the twenty imputed datasets.

- Model selection: LASSO

Lasso is a regression analysis using a shrinkage. LASSO regularization sets less-important predictors to 0 and helps in choosing the predictors that can be left out of the model. You must set a so-called "meta-parameter" (lambda) that defines how the aggressive regularization is performed. We choose the meta-parameter by cross-validation. We got two values of lambda, one that gave us the minimum mean cross-validated error and the largest value such that error is within one standard error of the minimum. We ran LASSO regression with 10 values of lambda between the minimum and maximum, and we fitted a model for each variable selection (for each lambda). Finally, we chose the most parsimonious model also considering clinical judgement. We repeated the process for the twenty imputed datasets.

Table S3. Models using LASSO method in one random imputed dataset.

Variables	Models									
	s0	s1	s2	s3	s4	s5	s6	s7	s8	s9
Intercept	-0.046	-0.334	-0.641	-0.962	-1.301	-1.660	-2.011	-2.367	-2.690	-2.881
Gender - Women	0.660	0.648	0.636	0.624	0.613	0.601	0.584	0.559	0.532	0.504
Gender – Transgender	0.618	0.599	0.580	0.562	0.542	0.523	0.496	0.466	0.435	0.406
Age - >60									0.061	0.158
Level of education – Higher education									-0.028	-0.055
Occupation – Not currently working									0.001	0.039
Occupation - Be on leave										0.044
Income - <1000	0	0.039	0.079	0.119	0.162	0.206	0.253	0.306	0.349	0.354
Income - 1001-2000									-0.017	-0.072
Income - >2001										-0.082
Alcohol dependence - High risk drinker										-0.030
Diagnosed in the last 12 months - Yes										0.087
Sexualized drug use – Once in a lifetime							-0.047	-0.117	-0.191	-0.270
Serologic disclosure - All or almost all								-0.050	-0.113	-0.176
Social isolation	0.032	0.034	0.035	0.036	0.038	0.039	0.041	0.043	0.045	0.047
Satisfaction with social role	-0.133	-0.133	-0.133	-0.133	-0.133	-0.132	-0.133	-0.133	-0.134	-0.135
Hours dedicated to leisure								-0.001	-0.003	-0.005

Quality of life – physical	0.025	0.026	0.028	0.030	0.032	0.035	0.037	0.040	0.042	0.044
Quality of life - mental	0.070	0.072	0.075	0.078	0.082	0.085	0.089	0.093	0.098	0.102
Cognitive function	-0.049	-0.05	-0.051	-0.053	-0.054	-0.056	-0.057	-0.059	-0.062	-0.065

- Patterns of substance use using Latent class analysis (LCA)

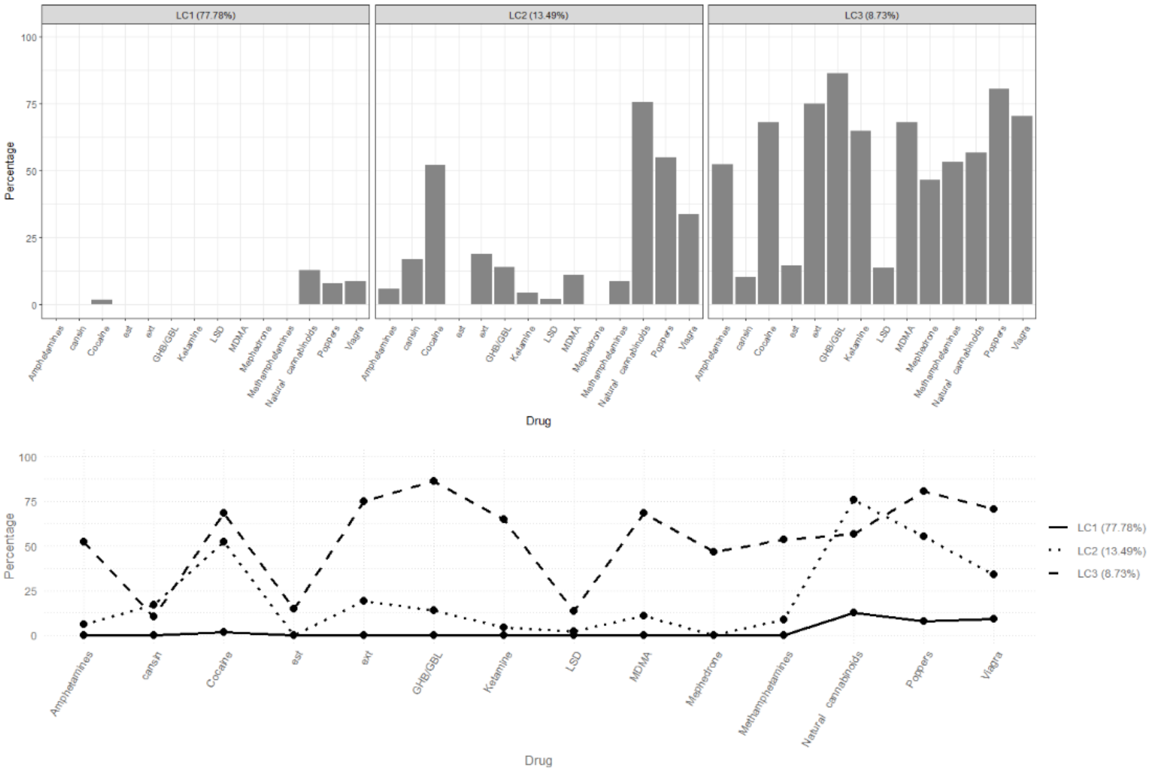

	1	2	3
0	565	0	0
1	187	1	0
2	32	49	0
3	0	52	0
4	0	21	6
5	0	10	13
6	0	3	16
7	0	0	13
8	0	0	11
9	0	0	8
10	0	0	6
11	0	0	9
12	0	0	3
13	0	0	3

Figure S1. Patterns of substance use and polyconsumption.

Note. Latent classes of substance use are presented in both figures, showing the proportions of use for each substance, according to patterns of substance abuse. The table represents the number of substances reported by the individuals classified in each of the three latent classes.
